# Effect of depth information on multiple-object tracking in three dimensions: A probabilistic perspective

**DOI:** 10.1371/journal.pcbi.1005554

**Published:** 2017-07-20

**Authors:** James R. H. Cooke, Arjan C. ter Horst, Robert J. van Beers, W. Pieter Medendorp

**Affiliations:** 1 Radboud University, Donders Institute for Brain, Cognition and Behaviour, Nijmegen, The Netherlands; 2 Vrije Universiteit Amsterdam, MOVE Research Institute, Amsterdam, The Netherlands; Queen's University, CANADA

## Abstract

Many daily situations require us to track multiple objects and people. This ability has traditionally been investigated in observers tracking objects in a plane. This simplification of reality does not address how observers track objects when targets move in three dimensions. Here, we study how observers track multiple objects in 2D and 3D while manipulating the average speed of the objects and the average distance between them. We show that performance declines as speed increases and distance decreases and that overall tracking accuracy is always higher in 3D than in 2D. The effects of distance and dimensionality interact to produce a more than additive improvement in performance during tracking in 3D compared to 2D. We propose an ideal observer model that uses the object dynamics and noisy observations to track the objects. This model provides a good fit to the data and explains the key findings of our experiment as originating from improved inference of object identity by adding the depth dimension.

## Author summary

Many daily life situations require us to track objects that are in motion. In the laboratory, this multiple object tracking problem is classically studied with objects moving on a two-dimensional screen, but in the real world objects typically move in three dimensions. Here we show that, despite the complexity of seeing in depth, observers track multiple objects better when they move in 3D than 2D. A probabilistic inference model explains this by showing that the association of noisy visual signals to the objects that caused them is less ambiguous when depth cues are available. This highlights the role that depth cues play in our everyday ability to track objects.

## Introduction

Throughout daily life we need to monitor and track our surroundings, avoiding collisions while walking, cycling or driving. This ability is based on estimating our self-motion and the motion of objects around us using visual cues such as retinal motion, binocular disparity, relative size and motion parallax. A complexity arises because these cues are noisy and often ambiguous; for example, both a moving object and an eye movement create retinal motion. To form inferences about how objects move in the world around us, the brain must therefore disambiguate cues and integrate noisy information. Here, we focus on the complexities involved in tracking with multiple moving objects.

Within the laboratory, our ability to track moving objects is typically investigated using a multiple object tracking (MOT) paradigm, in which a subject tracks a subset of targets out of a larger number of objects as they move on a 2D screen. These experiments have a long history showing that many factors influence our tracking capability. Tracking accuracy appears to decline with increasing object speed [1], number of objects [2] and the relative closeness of objects [3], but can be increased by simply coloring objects differently [4] or altering object shapes [5].

Although these findings may seem disparate, Vul et al [6] have recently provided a normative explanation. The authors view multiple object tracking as a data association or correspondence problem, referring to a problem that broadly needs to be solved in cognitive behaviors, such as in the matching of binocular images for stereovision or to prevent multiple items to be swapped when stored in memory [7,8].

Vul and colleagues modeled object tracking by devising an ideal observer model in which the uncertainty of position and velocity signals affects how well these signals can be associated to the objects that caused them. In the model, the position uncertainty increases with number of tracked objects, similar to other suggestions [9]. As a result of this uncertainty, noisy position measurements cannot distinguish between objects if these are close together. The model, however, also uses velocity signals to distinguish between objects, which is especially useful when they are close. However, as objects move faster, the velocity measures will become more uncertain, so that at high velocities, the ability to distinguish between objects will decline and the predictions about their future position will deteriorate. This causes performance to decline as objects move closer together and as they move faster.

While multiple object tracking studies generally focus on objects moving in the two-dimensional frontoparallel plane, this is an atypically simple, special case. In real life, objects move continuously in all three dimensions [10–14]. If multiple object tracking reflects an association problem, then adding depth information may promote tracking. More specifically, two objects that move closely together from a two-dimensional frontoparallel perspective but far apart in depth, may still be correctly associated using depth cues. Indeed, Ur Rehman, Kihara, Matsumoto, & Ohtsuka [15] already reported that tracking performance improves when the moving objects are separated by moving in different depth planes. But, again, during realistic 3D object motion objects are not restricted to moving only in different depth planes. How is tracking performance affected when realistic depth cues and continuous motion in depth are present?

Thus far, only few studies have performed a direct comparison between object tracking in 2D and 3D. Both Liu et al [16] and Vidakovic & Zdravkovic [17] added monocular pictorial depth cues to the scene, but found no significant improvement in object tracking, suggesting that such cues are not precise enough to help solve the correspondence problem. Of course, this cannot be generalized to all depth cues. For example, binocular disparity is the main binocular cue for depth, and known to be more reliable than pictorial cues [18].

In this study, we investigated how tracking performance changes when objects move in continuous 3D space (displayed using both monocular and binocular cues) compared to moving in a single depth plane. To assess the role of depth information, we manipulated the average speed and average distance between the objects in all dimensions. Following Vul et al [6], we constructed four versions of an ideal observer model to test how position and velocity information could be incorporated into multiple object tracking in 3D.

## Methods

### Participants

Ten healthy naïve subjects (8 female), aged 18–30 years, participated in this study. All subjects had normal or corrected to normal vision, including normal stereovision (tested using the Randot Stereo test (Stereo Optical Inc., Chicago, USA)) and no known history of neurological or visual disorders. Informed written consent was obtained from all subjects prior to the experiment and the experiment was approved by the Ethics Committee of the Faculty of Social Sciences. One subject failed to comply with the task instructions, and was removed from the subject pool.

### Apparatus

Visual stimuli were projected using two digital stereo DLP®-rear projection cubes (EC-3D-67-SXT+ -CP, Eyevis GmbH, Reutlingen, Germany) on a 2.83 X 1.05 m (width X height) surface with a resolution of 2800 by 1050 pixels. Subjects were seated 1.75 m in front of the center of the screen, which thus subtended 77.9° X 33.4° of visual angle. Vertical retraces of the images were synchronized using an Nvidea Quadro K5000 graphics card. The visual display was updated at 60 Hz. Stereoscopic images were generated using channel separation, based on interference filter technology (INFITEC® GmbH, Ulm, Germany), projecting images for the left and right eye using different wavelengths. Subjects wore a pair of glasses with selective interference filters for each eye and used a chin rest for stabilization.

### Stimuli

Visual stimuli (referred to as objects from now on) consisted of spheres shaded to appear 3D. The shading was constant across objects and depth, which prevented it being used to discriminate different objects. The objects subtended 0.5° visual angle at screen depth and were rendered in a virtual space of 3.00 m wide, 2.00 m high, and 1.75 m deep (0.875 m in front and 0.875 m behind the screen) using their 3D position. The visual scene also contained a stationary yellow fixation cross of 0.2° visual angle at screen depth straight ahead of the observer. Objects were rendered in OpenGL using a realistic perspective transformation, thus providing multiple depth cues such as relative size, motion parallax and binocular disparity.

During each trial the position of the objects was updated according to a modified Ornstein-Uhlenbeck process, as used by Vul et al [6]. Objects moved according to Brownian motion while being attached to a virtual spring situated at zero (where zero is the center of the display):

$$x_{t}=x_{t-1}+v_{t}$$ (1)

$$v_{t}=\lambda v_{t-1}-kx_{t-1}+w_{t}$$ (2)

$$w_{t}∼N(0,\sigma_{w}^{2})$$

in which *x*_*t*_, *v*_*t*_, *w*_*t*_ are the position, velocity and random acceleration of the object at time step *t*, respectively. *k* is a spring constant which was varied to generate desired dynamics and *λ* is a damping term which was fixed to 0.9. These dynamics cause the objects’ position and velocity to evolve stochastically but allow their variances to be expressed in closed form. This enabled us to systematically manipulate how close the objects were to each other on average, and how fast they moved on average. Specifically, we calculated the spring constant *k* and the acceleration variance $\sigma_{w}^{2}$, to produce a desired *σ*_*x*_: the standard deviation in object position and *σ*_*v*_: the standard deviation of their velocity. This was done by assuming that these variances do not change across time steps. The stationary standard deviations of position and velocity of an object are as follows:

$$\sigma_{x}=\sqrt{\frac{(1+\lambda )\sigma_{w}^{2}}{k(\lambda -1)(k-2\lambda -2)}}$$ (3)

$$\sigma_{v}=\sqrt{\frac{2\sigma_{w}^{2}}{\left(\lambda -1)(k-2\lambda -2\right)}}$$ (4)

These equations can be rearranged to calculate the spring constant and acceleration variance required to produce a desired *σ*_*v*_ and *σ*_*x*_.

$$\sigma_{w}^{2}=\frac{\left(\lambda^{2}-1)\sigma_{v}^{2}(\sigma_{v}^{2}-4\sigma_{x}^{2}\right)}{4\sigma_{x}^{2}}$$ (5)

$$k=\frac{(1+\lambda )\sigma_{v}^{2}}{2\sigma_{x}^{2}}$$ (6)

We used the same dynamics but independent noise for each dimension of object motion. For the frontoparallel plane these calculations were performed in visual angle and then converted into meters for display. The same value in meters was used for the depth dimension.

### Procedure

**Fig 1:**
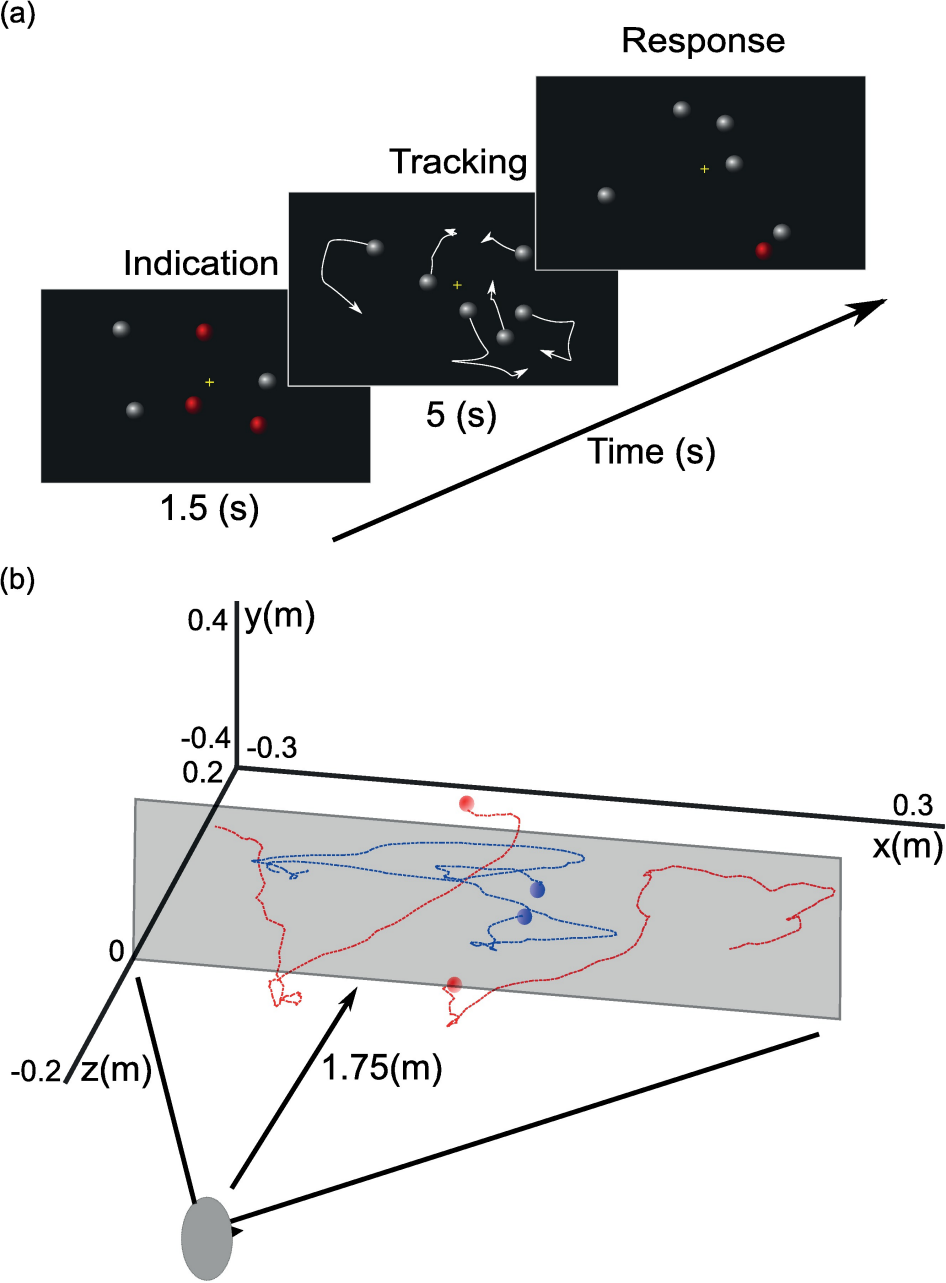
Schematic representation of multiple object tracking task. (a) Multiple object tracking. Subjects tracked three indicated objects before responding whether or not a probed object was a target. (b) Example trajectories of four objects through virtual space. Dashed lines indicate object trajectories over 1.5s, disks indicate trajectory start and the grey plane indicates the screen. The blue and red lines represent part of the 2D and 3D trajectories, respectively. Trajectories were taken from trials with *σ*_*x*_ = 4° and *σ*_*v*_ = 0.2° per frame.

The subject had to track three out of six moving objects (see Fig 1). Each trial began with the presentation of six stationary objects for 1.5 s. Three of these objects were white and the ones that had to be tracked were cued red. Next, all objects turned white and began to move according to the dynamics described above. The subject had to track the cued objects for 5 s after which all objects stopped moving and one was randomly turned red. The subject had to indicate if this was one of the originally cued objects, using a button press. Then the next trial began.

The initial positions and velocities of the objects were randomly sampled using the position and velocity standard deviation for that trial, i.e., *σ*_*x*_ and *σ*_*v*_, respectively (see Table 1). Furthermore, objects moved either in 2D (frontoparallel plane) or in 3D. Each subject was tested in 60 conditions, split into 6 sessions of 45 minutes, the order of which was counterbalanced across subjects. In each session, we fixed the value of *σ*_*x*_ to reduce performance effects caused by estimation of the movement dynamics. Within each session, *σ*_*v*_ was randomly selected on each trial from a set of fixed values (see Table 1) and 30 trials were performed for each value. Prior to each session, subjects performed 15 practice trials, leading to a total of 1890 trials (6 sessions * 10 parameter values * 30 trials + 6 practice blocks *15 trials).

**Table 1:** Experimental sessions with *σ*_*x*_, *σ*_*v*_, and depth conditions.

Exp	*σ*_*x*_(°)	*σ*_*v*_(°/frame)	Dim
**1**	2	0.005, 0.02, 0.035, 0.05, 0.065, 0.08, 0.1, 0.12, 0.156, 0.2	2D
**2**	3	0.005, 0.0267, 0.0483, 0.07, 0.0917, 0.1133, 0.135, 0.1567, 0.1783, 0.2	2D
**3**	4	0.005, 0.0267, 0.0483, 0.07, 0.0917, 0.1133, 0.135, 0.1567, 0.1783, 0.2	2D
**4**	2	0.005, 0.02, 0.035, 0.05, 0.065, 0.08, 0.1, 0.12, 0.156, 0.2	3D
**5**	3	0.005, 0.0267, 0.0483, 0.07, 0.0917, 0.1133, 0.135, 0.1567, 0.1783, 0.2	3D
**6**	4	0.005, 0.0267, 0.0483, 0.07, 0.0917, 0.1133, 0.135, 0.1567, 0.1783, 0.2	3D

### Analysis

Data were analyzed using Matlab 2014b (The MathWorks, Natick, MA, USA). To assess how tracking accuracy changed as a function of *σ*_*v*_, we fit a psychometric curve to the proportion of correct responses for each session. Because of asymmetry in the data we used a cumulative Weibull distribution:

$$p=g+(1-g-\gamma )*(1-e^{-{\left(\frac{1}{{a\sigma }_{v}}\right)}^{\beta }})$$ (7)

in which *p* is the proportion of correct responses, *g* is the guess rate, *γ* is the lapse rate, *σ*_*v*_ is the velocity standard deviation of the trial, *a* is the scale parameter and *β* is the shape parameter. Because a Weibull distribution requires *β* > 0, we used $\frac{1}{\sigma_{v}}$ as the stimulus because this co-varies positively with performance.

We fit the parameters of the psychometric function to the data of each subject and session separately, allowing the scale, shape, and lapse rate to change across sessions and subjects. Fitting was performed using a maximum likelihood approach by computing the probability of each response given the parameter values and finding the parameter values that maximized this probability. Furthermore, *γ* was constrained between 0 and 0.2 and *g* was fixed to 0.5 in the fitting procedure.

To measure the effect of distance and depth cues we compared the fitted psychometric curves by inverting the Weibull function to identify the velocity standard deviation *σ*_*v*_ value that would yield a particular correct response probability.

$$\sigma_{v}=\frac{1}{a*{\log\left(\frac{\gamma +g-1}{\gamma +p-1)}\right)}^{\frac{1}{\beta }}}$$ (8)

For our comparisons, we used the 0.75 proportion correct as criterion level of performance. These values were submitted to a within-subject analysis of variance (ANOVA) to assess the influence of spatial extent (three levels: *σ*_*x*_ = 2, 3, and 4°) and dimensionality (two levels: 2D and 3D).

### Model

Vul et al [6] described and used a Bayesian tracking solution for multiple object tracking in 2D. Here we used and expanded this modeling approach to account for object tracking in 3D, in which depth information is added to the model and used to resolve uncertain data associations. In the model, we assume the observer represents the objects by their position and velocity in 3D, that is a position and velocity state for x, y and z (i.e., depth) dimensions (see Fig 1). We used meters and meters per frame for the position and velocity units, respectively.

Given the linear Gaussian dynamics of the objects and noisy observations, we estimated the state of each object using a Kalman filter. This is an approximation since the Kalman filter is a suboptimal estimator when the noise in the measurements is state dependent (see below). However, the difference between the distributions is small and this approximation allows us to maintain analytical tractability.

The Kalman filter incorporates two sources of noise, process noise, which is part of the object dynamics, and measurement noise, which arises in the observer during observation of the stimuli. The variance of the process noise is given by $\sigma_{w}^{2}$ (see Eq 5). The measurement noise is specified by the sensory noise of position and velocity in each dimension. It is assumed that position noise in the frontoparallel plane (x and y axis) increases with eccentricity [19].

$$\sigma_{p_{x}}=c(1+14|p_{x}|)$$ (9)

$$\sigma_{p_{y}}=c(1+14|p_{y}|)$$ (10)

in which c is a free scaling parameter and *p*_*x*_ and *p*_*y*_ are the x and y position of the object in meters relative to the fixation point. The depth noise follows from stereoscopic uncertainty, which is known to modulate as a function of retinal eccentricity [20] and distance from fixation in depth [21]. We converted the scaling factors found in these studies into meters yielding:

$$\sigma_{p_{z}}=d\left(1+14\sqrt{p_{y}^{2}+p_{x}^{2}}\right)(1+1.5|p_{z}|)$$ (11)

where d is a free scaling parameter for our stimuli and *p*_*z*_ is the position along the depth axis (z axis) with zero at the fixation point, which is at the center of the screen.

For modeling the velocity noise in the frontoparallel plane (x and y axis), we used Weber scaling [22].

$$\sigma_{vx}=0.05|v_{x}|$$ (12)

$$\sigma_{vy}=0.05|v_{y}|$$ (13)

Finally, the model takes the noise in the stereomotion signals into account. Based on Cumming [23] we assume a linear relationship between stereoacuity and stereomotion thresholds, with a slope of about 1.66. As a result, the standard deviation of velocity noise in the depth direction was taken as.

$$\sigma_{vz}=\sigma_{p_{z}}1.66$$ (14)

Given the above measurement equations and the dynamics described in the stimuli section, we used the Kalman filter to estimate the state of a single object (see S1 Text). Because multiple objects must be tracked, there is an additional complexity for the model, i.e. which measurement to use to update the state of which object? The exact Bayesian solution to this problem is to estimate the state of each object given every measurement and then to sum the state estimates based on how likely this assignment is. This is computationally demanding given that the six objects in our task yield 720 possible permutations of assignments at each time step.

In the model, this is resolved by selecting the assignments based on their probability [6]. Using the Kalman filter approach, the probability that a perceptual measurement originated from a particular object can be computed in closed form, which indicates how likely each permutation of assignments is. The model selects the three assignments with the highest probability and computes the state estimate based on them [24,25]. See S1 Text for full description of the tracking algorithm.

**Fig 2:**
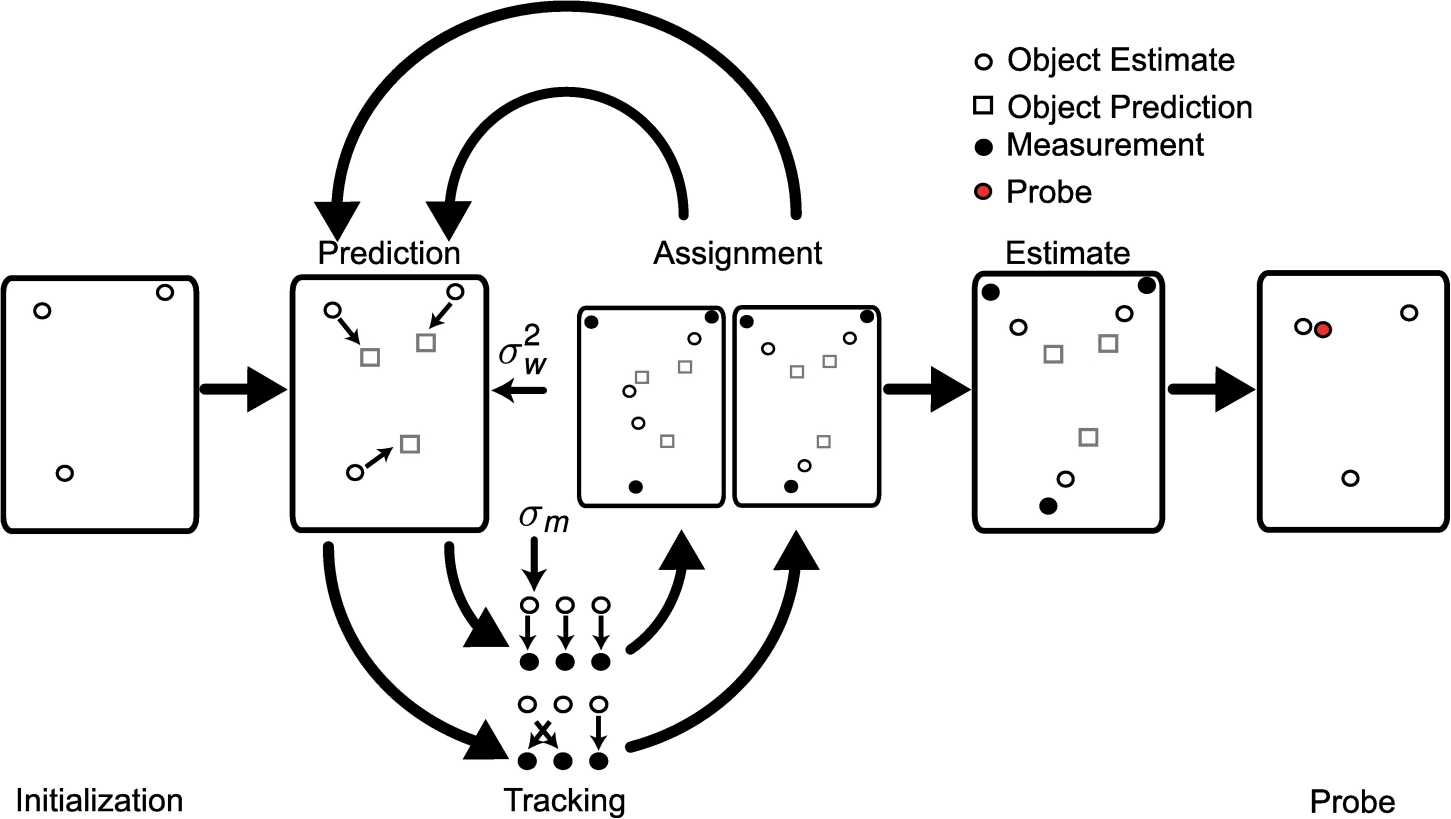
Schematic representation of ideal observer MOT model. White circles indicate the model’s estimate of an object state; black circles indicate their noisy perceptual measurements (indicated by *σ*_*m*_). Grey squares indicate the predictions of their future state. $\sigma_{w}^{2}$ represents the process noise variance used for prediction. The initial model estimates are set to the objects’ start position. The model proceeds to track the objects for the duration of the trial. This is accomplished by predicting the future state using the process dynamics and combing this with noisy perceptual measurements to determine the measurement assignments. The assignments are used to estimate the objects’ state. The estimates are used to generate predictions and the process is repeated until the end of a trial. At the end of a trial the model is probed to test if objects were correctly tracked. This was done by drawing a random sample from the position of one object and calculating the probability this sample came from a target or non-target.

The model uses three data assignment vectors at each time step, following previous sample based models [26–28]. The model simulated 1000 trials for each of the conditions subjects underwent. Each trial consisted of three main phases. First, the model was provided stationary objects to initialize the state estimates without velocity information. Secondly, the model tracked the moving objects for the same duration as the human observers using noisy perceptual measurements of the true states. Finally, we drew a sample from the final state of one object (the probe), and corrupted it with additive measurement noise according to the above equations and computed the probability of this belonging to the estimates of each object. The model responded the probe was from a target if the sum of the target probabilities exceeded that of the non-targets. A schematic illustration of the model can be seen in Fig 2.

### Model versions

The model tracks the objects based on the perceptual signals it has available. Because there is no consensus in how velocity information is incorporated into object tracking [6,29,30], we considered four variants of our model.

First, we tested the full extrapolation model (FE model). This is the most complete version of the model, as described above, predicting the objects future positions using the process dynamics that generated the object trajectories (see Stimuli). Hence, this model represents an observer who knows, according to the dynamics, how the objects move, thereby combining extrapolation and noisy perceptual measurements.

Second, we considered two models without the velocity-based extrapolation step (NE models). These versions of the model represent an observer who perceives velocity and uses it to dissociate assignments, but does not use the velocity information for extrapolation. We implemented this as follows. In one version, *λ* was set to 0.9 in Eqs 5 and 6 and 0 in Eq 2, thereby representing an observer with correct knowledge of the object dynamics but without extrapolation (NE-cd). In the other version, we set *λ* to 0 in Eqs 2, 5 and 6, which means that the observer does not extrapolate, as for the NE-cd model, but also uses incorrect dynamics for tracking (NE-id).

Finally, we considered a version of the model in which tracking is based on position measurements only, without involving velocity information in any way (No Velocity (NV) model). In this version of the model, we removed the velocity states from the Kalman filter, thereby modeling an observer who did not use velocity at any point in the tracking process.

**Fig 3:**
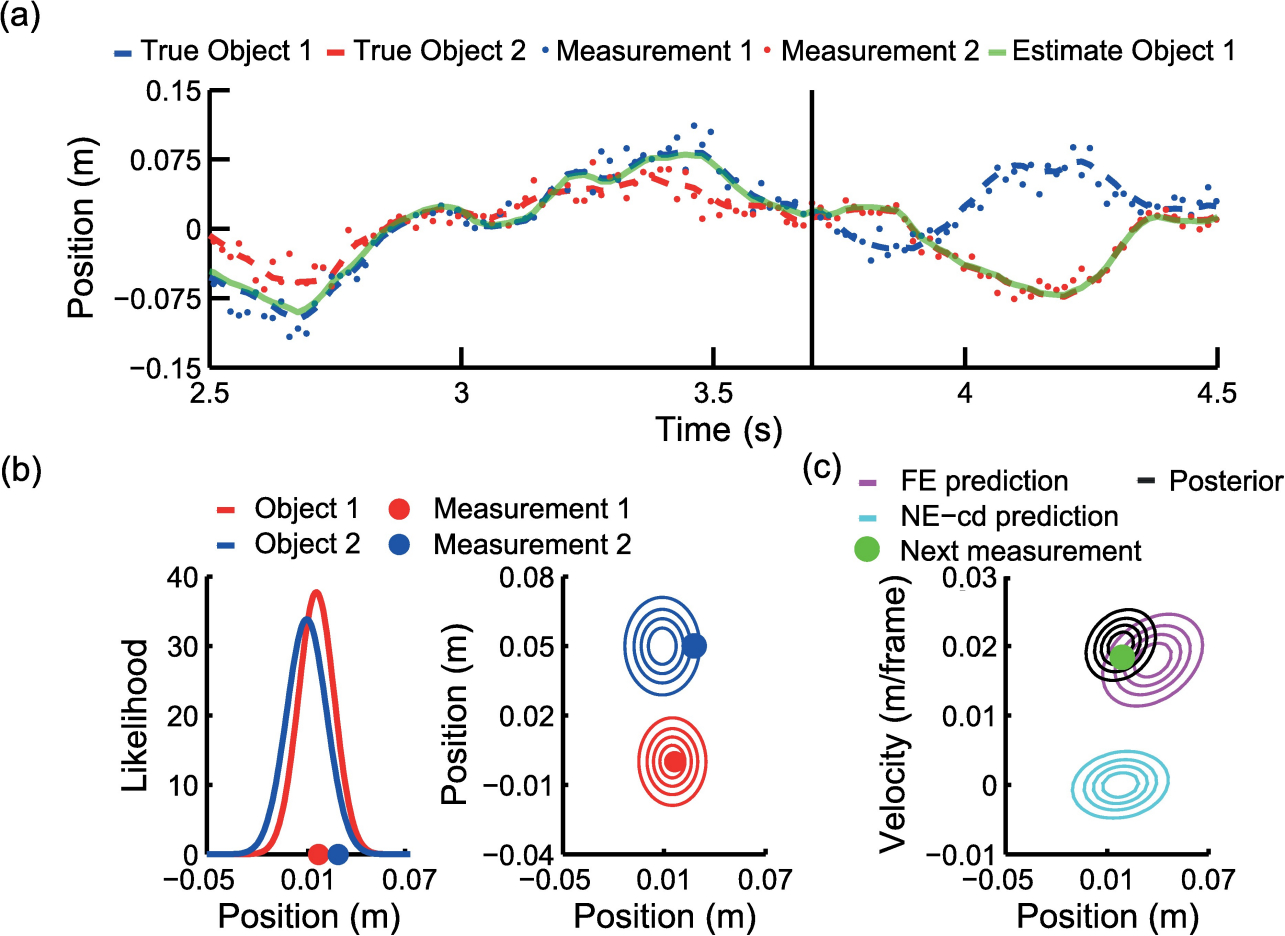
Simplified model illustration. (A) NE-cd model run on object trajectories for two objects, including confusion of objects. The red and blue dashed lines represent the actual trajectory for two different objects, the green line indicates the model’s position estimate of one object. The vertical black line indicates the time point of the data used in B. (B) Likelihood of a measurement coming from object 1 (blue) and object 2 (red) in the 2D case (left) and 3D case (right). Contour plots represent four slices of the two-dimensional likelihood function, evenly spread from the minimum to maximum likelihoods. Similar likelihoods in one dimension can be disambiguated in the other dimension. (C) Contour plots of predicted state and covariance given the posterior distribution of previous time step (black) for FE (magenta) and NE-cd (cyan) model, NE-cd does not use velocity information in the prediction, leading to biases towards zero velocity and position. Data was generated with *σ*_*x*_ = 2° and *σ*_*v*_ = 0.2° per frame using the best fit parameters from the NE-cd model (see Table 2).

For illustration purposes, Fig 3A shows a simulation of the NE-cd model in a simplified tracking task with two objects. As shown, during the tracking, the model initially tracks the position of each object quite accurately but at about 3.7 s, a point of confusion occurs and the model swaps the two objects in the further tracking. Fig 3B, which shows the likelihood of the measurements arising from each object at the point of confusion, suggests that measurements in this case are more likely to come from the other object. Depth information may improve tracking by making this association problem easier, as illustrated in Fig 3B. As shown, a measurement that would incorrectly be assigned in one dimension, may be correctly assigned using the information from the additional dimension. In other words, the additional dimension helps to correctly infer which object generated the measurement, thus disambiguating the assignment. Of note, this disambiguation not only depends on the dimensionality of the task but also how well the future positions of the objects can be predicted.

Fig 3C illustrates the predictions of the FE and NE-cd model. In contrast to the FE model, in the NE-cd model current velocity does not influence the position and velocity estimate at the next time step. Not using velocity information causes a bias towards zero velocity at the next step. A bias towards zero position is also seen due to the spring dynamics used. Accordingly, it is more difficult to accurately predict the motion of the objects and therefore assign the perceptual measurements correctly.

### Model fitting

In the model, parameters c and d are free scaling parameters. We fit these parameters to the pooled group data using a maximum likelihood approach. The fit procedure was performed by finding the values that maximize the likelihood of the data given our model. As the data takes the form of a discrete number of correct answers for each of the 60 conditions, we computed the log likelihood of our data given the model as

$$\log L(\left\{c_{i}\right\}|model)=\sum_{i=1}^{60}log(B(c_{i};N_{i},p_{i}))$$ (15)

where B is a binomial distribution evaluated for each condition *i* with the number of correct responses *c*_*i*_, number of trials *N*_*i*_ and the proportion correct of the model *p*_*i*_ as the probability.

## Results

### Psychometric results

**Fig 4:**
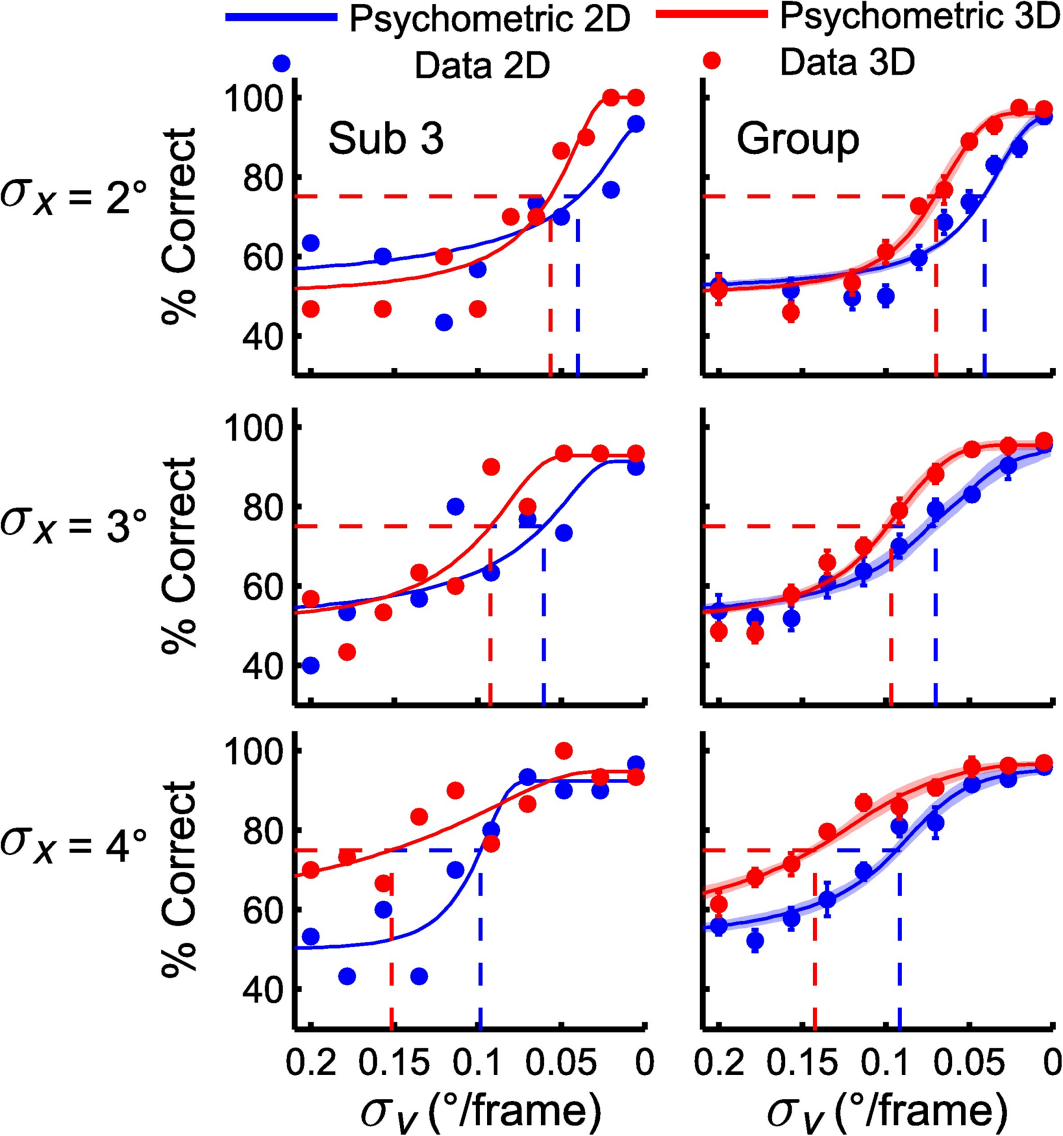
Accuracy data from tracking experiment. Data and fitted psychometric curves for a single subject (left) and group data (right). Data points indicate percentage of correct responses. Error bars indicate 1 standard error calculated across subjects. Shaded areas indicate 1 standard error of psychometric curves across subjects. Dashed lines indicate *σ*_*v*_ value for 75% correct performance used for comparison across conditions.

The left panels of Fig 4 show the results of a typical subject when objects were tracked in either 2D (in blue) or 3D (in red). Data points indicate the percentage of correct responses as a function of the velocity standard deviation (*σ*_*v*_), for the three values of the position standard deviation (*σ*_*x*_). Note the reversed velocity axis (abscissa)–the origin is on the right of the x-axis. When objects move at the highest average speed (*σ*_*v*_ = 0.2°/frame), the subject reports at chance level (50% correct), while for lower speeds tested (*σ*_*v*_ < 0.03°/frame) performance is nearly perfect, irrespective of the position variance. We fitted psychometric curves through these data (see Methods, Eq 7).

As a performance threshold we took the velocity standard deviation at which the subject responds in 75% of the trials with a correct answer. As shown, performance thresholds are higher when objects move in 3D than in 2D (red curve are leftward shifted relative to the blue curves) and are also increased in the sessions with higher position variance. This suggests that this subject could track objects at a higher speed when the mean distance between the objects increased and when depth information was added.

The results of this subject are exemplary for all subjects. Their average data and fitted curves are shown in the right panels of Fig 4. The 2D results are consistent with the observations of Vul et al [6], tracking accuracy declines as speed increases but increases with distance between objects. The 3D results show that adding depth information improves tracking performance.

**Fig 5:**
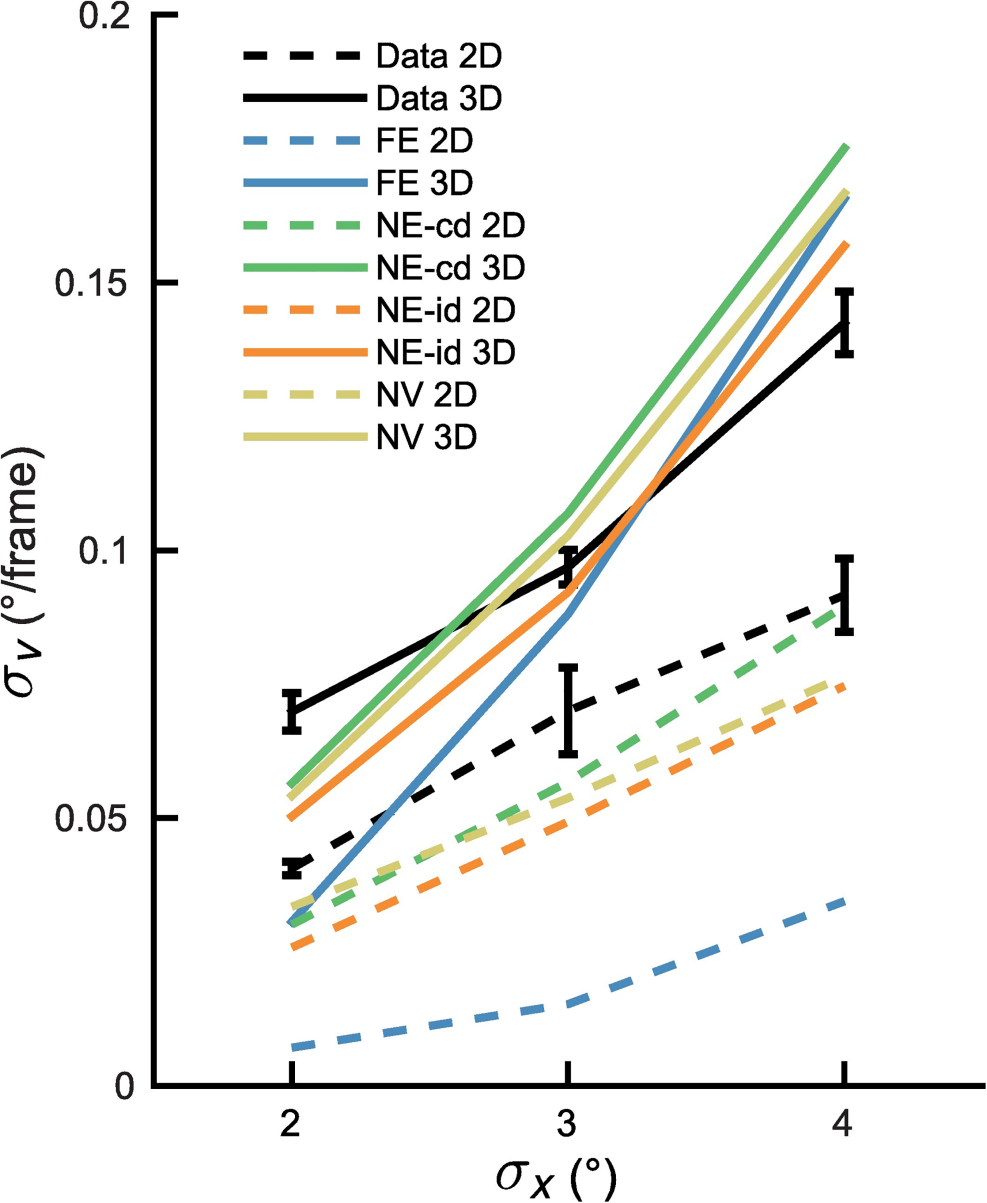
Distance and depth interaction. Interaction between depth and distance for the *σ*_*v*_ value required for 75% correct performance in 2D and 3D conditions for the different models. Solid lines indicate 3D conditions, dashed lines the 2D conditions, black lines indicate data and the colors represent model predictions (Blue: FE, green: NE-cd, orange: NE-id and yellow: NV). Error bars indicate one standard error.

Threshold values were extracted based on the individual fits, then averaged, and plotted in Fig 5 as a function of position standard deviation (black dashed and solid lines). A within-subject ANOVA with position standard deviation (three levels: *σ*_*x*_ = 2, 3 and 4°) and dimensionality (two levels: 2D and 3D) as factors revealed not only significant main effects of position standard deviation (*F*(2, 16) = 78.52, *p* < .001) and dimensionality (*F*(1,8) = 151.07, *p* < .001), but also a significant interaction (*F*(2,16) = 5.20, *p* = .018). Posthoc testing showed the difference between 2D and 3D tracking is significant for all three *σ*_*x*_ values (paired t-tests, *p*<0.01). Thus tracking performance is better when objects are further apart not only in 2D but also 3D, with the depth interacting to produce a more than additive effect on performance.

### Model predictions

**Fig 6:**
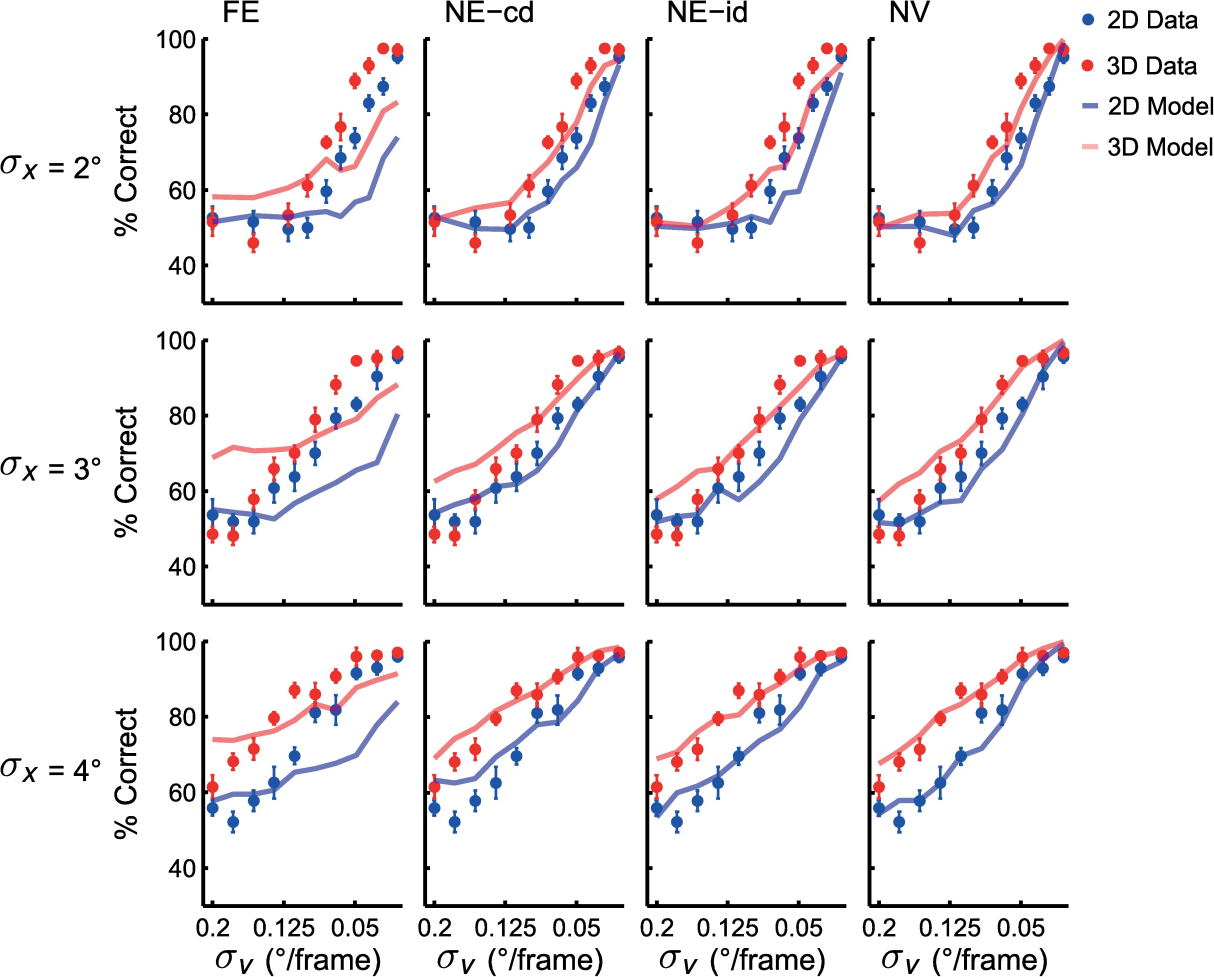
Model predictions. Data points indicate percentage of correct responses for this stimulus combination. FE is full extrapolation model, NE-cd is the no extrapolation with correct dynamics model, NE-id is the no extrapolation with incorrect dynamics model and NV is no velocity model. Blue and red lines indicate percentage correct predictions for 2D and 3D conditions, respectively. Error bars indicate one standard error.

In order to account for the data, we specified four versions of the optimal observer model for object tracking in 2D and 3D. The model versions differ as to how the velocity information is taken into account by the observer. More specifically, the FE model tracks objects optimally by combining extrapolation with noisy position and velocity measurements. The NE models obtain noisy perceptual measurements of velocity information without using this information for extrapolation and the NV model does not take velocity information into account at all. The colored lines in Fig 6 present the predictions of the four model versions together with the subject data. The FE-model does not capture the data well, while the NE-id, NE-cd and NV-models perform reasonably well. This can also be seen in the predicted velocity standard deviation thresholds shown in Fig 5, where the FE model underestimates some thresholds while overestimating others in 3D and underestimates them in 2D. To perform a quantitative comparison of the models, we computed the relative log likelihood of each model (compared to most likely model) given our data and the best fit parameters (see Table 2). The relative log-likelihoods show evidence in favor of the NE-cd model. Note, as all models include the same number of parameters, corrections such as AIC or BIC are not required for model comparison [31]. It should be noted that for computational reasons these fits were obtained through a rough grid search and as such slightly better fits may be obtainable. We verified for each model that the likelihood function had a concave shape for the grids used. Therefore, the minima of each should be a reasonable representation of the parameters.

**Table 2:** Maximum Likelihood parameters and quality of fit for the four models.

Model	c (m)	d (m)	Relative log likelihood
**FE**	0.020	0.0169	-580.74
**NE-cd**	0.0082	0.0202	0
**NE-id**	0.010	0.0250	-32.80
**NV**	0.0014	0.0101	-69.91

c is a free scaling parameter for position noise in the frontoparallel plane, d is a free scaling parameter for position noise in the depth plane.

## Discussion

We show that objects that move in 3D are tracked better than objects moving in 2D and that the magnitude of this improvement increases with the mean distance between objects in 3D space. We compared four ideal observer models to aid in providing a quantitative explanation behind these results. We find an ideal observer model that tracks objects optimally (FE-model) by extrapolating the next position and combining this with noisy perceptual measurements cannot account for our behavioral data. Instead models that assume subjects use velocity information suboptimally (NE-cd, NE-id, and NV models) provide a better fit to the data. Specifically, we find that our No Extrapolation with correct dynamics (NE-cd) model, which receives velocity measurements from the objects but cannot use velocity to predict the next position, provides the best fit.

The NE-cd provides an intuitive explanation for the benefit of depth information. When we track multiple objects we must infer which objects generated which noisy perceptual measurements to both identify the targets and to generate accurate predictions. As we mentioned previously, inferring the correct data assignments is easier when we have the additional depth dimension because assignments that can be confused in 2D are likely to be disambiguated in 3D. The interaction between depth and distance can be explained in a similar manner. Although having an additional dimension provides the capability to disambiguate which objects generated which measurements, the distance between the objects in this additional dimension is crucial. If objects are close together in depth then perceptual noise could still cause objects to be confused. As we increase the distance we reduce the overlap between the predictions of one object and the measurements of another making it more difficult to confuse them. As such, our model can explain our finding that increasing speed lowers tracking performance because it increases our uncertainty [6] and depth improves tracking by also making it harder for predictions and measurements to overlap. In addition, our model also allows us to explain why objects placed in depth planes are tracked more accurately [15,32]. Placing objects in different planes disambiguates object to measurements assignments when they are close together in 2D thereby reducing the number of incorrect assignments.

Additionally, if our model is a realistic approximation to the task then the noise parameters obtained after fitting should be consistent with other work. Indeed, the frontoparallel noise scaling (c) and depth noise scaling (d) of the best-fit model are similar to those previously reported. Bayes & Husain [33] found the precision of positional short term working memory for 3 items to be approximately 0.5 deg⁻¹ with the items being shown 10 deg to the left of fixation. Using Eq (9) and converting to their units produces an estimated precision from our model of 0.7 deg⁻¹. For eccentricity scaling of depth noise our model predicts a standard deviation of 0.0202 m at fixation and 0.0981 m at 9 deg, similar to previously reported values which were between 0.0087–0.0195 m in the fovea and between 0.0479–0.1831 m at 9 deg [20]. Although these tasks are different from ours they do illustrate the values obtained are plausible and within the range of previous data providing some additional support of our model.

Despite the NE-cd model successfully explaining our experimental observations there are still components of the tracking process that need further investigation. Firstly, the noise terms we use in our model are simplifications. Investigation into how realistic these simplifications are is needed. To illustrate this, the current model cannot explain the finding that tracking objects in two different planes is harder when the planes are separated by large distances compared to small distances [15]. One explanation is that the noise in our estimates of object position in the frontoparallel plane is affected by distance from fixation, a component that was not introduced into our model. However, it could also be that the additional distance alters the size and contrast of the retinal image thereby changing the perceptual uncertainty while maintaining the independence of frontoparallel noise and depth. Therefore, research is needed to investigate how distance from fixation affects tracking in a virtual rather than real 3D set up where these properties can be tightly manipulated. Secondly, we only compare four possible models for velocity usage, one of which predicts the next position of the object and combines this with noisy measurement (FE), two of which perceive velocity information but do not use it for prediction (NE models) and one of which uses only position information for all the tracking (NV). There are additional possibilities for how observers could use velocity information. It is possible that observers do extrapolate but that there is a difference between the true motion of the objects in experiments and the model used by subjects, an idea which has also been presented to explain findings in visual working memory [34]. Alternatively, individuals may not build models of object motion in tracking tasks, but instead make predictions only using perceived velocity and Newtonian dynamics [30]. This multitude of possibilities makes it difficult to draw too strong conclusions about the role of velocity information in MOT. However, as the perfect extrapolation model produced the worst fit it is evident that some form of under extrapolation is present. This is consistent with experiments showing that when objects are being tracked and become occluded, accuracy is higher if they reappear where they disappeared rather than at their extrapolated position [35,36].

An attractive way to investigate which sensory noise model and velocity model underlies our tracking ability would be to use factorial model comparison [37,38]. Essentially, this uses Bayesian model comparison [39,40] to compare sets of models. This could be used to investigate different noise models and different ways velocity is incorporated to identify which pairing best fits human tracking data. Unfortunately, modeling MOT is difficult as the task is inherently computationally intensive and model comparison requires thousands of iterations per model to integrate over the parameter space. As such it may be appealing to consider simpler tasks that still capture the elements of MOT to facilitate modeling attempts of the underlying processes. For example, Ma & Huang [9] modeled multiple trajectory tracking, a task in which observers see multiple dots moving left to right and have to report whether they deviated at the mid-point. This simple task embodies some elements of MOT such as the influence of sensory noise and solving the correspondence problem. It can be formulated in an analytical way to allow for efficient model comparison. However, this task may not be ideal to study the role of velocity information, as it does not require a large focus on extrapolation. A similar experiment that requires more positional extrapolation may prove useful to determine different noise models and how velocity is used. Additionally, in this experiment each object is relevant to the task, in contrast MOT tasks typically incorporate distracters, which may affect the tracking process.

Furthermore, we made the assumption that observers track both non-targets and targets identically. Other models have been proposed that exclusively track targets [30], however, there is experimental evidence that both targets and non-targets are tracked. That is, if subjects perform an MOT task and are asked to report when a probe is presented on a target or non-target they detect the probe more often on a target, but still detect it on non-targets as well [41]. This suggests observers track both targets and non-targets but not in identical ways. An additional extension to our model would be to consider modifications that allow tracking to differ between targets and non-targets while maintaining its current explanatory power.

Our model also has implication for future work in MOT. Specifically, it makes predictions about which factors should influence the difficulty of the assignment problem and therefore which factors should affect tracking performance. For example, our model predicts that the amount of facilitation that 3D motion provides is dependent on the precision of the depth information. If the precision of our depth estimate is low then the improvement should also be low and vice versa. This has been tested somewhat indirectly, as precise depth cues such as disparity alone can improve tracking performance [32] but less precise depth cues such as relative size do not [16]. We do not know of any experiments directly testing if gradual manipulations in depth cue reliability produce the expected effect. Our model also has implications for 2D MOT. Theories have proposed that tracking performance is limited only by the distance between objects, and not to the number of objects or speed [42]. Our model suggests it is not distance alone but the relationship between distance and measurement precision. This yields the experimental prediction that objects can be close together and are still trackable if measurement precision is high but creating poorer precision should require moving objects to be further apart to produce the same performance. To our knowledge there is no work testing the role of measurement precision on tracking in either 2D or 3D MOT. Doing so would greatly improve our knowledge of the role uncertainty plays in our capability to track multiple objects.

Due to the generality of the correspondence problem in visual perception and cognition, the finding that depth cues reduce correspondence errors has implications for other topics. For example, a significant source of errors within working memory experiments are so called “swap errors” [8]. These refer to errors in which an observer recalls not the item probed but another memorized item. It has been shown the number of these errors increases as objects are brought closer together [8]. This suggests that these errors result from making an incorrect correspondence between the location probed and the existing memory representation. Depth cues could play a role in reducing the occurrence of this type of errors within working memory. A recent change detection experiment provided some support for the idea that depth cues reduce swap errors [43]. In this experiment, subjects had to memorize a display of colored items whose position was either 2D or 3D. Subsequently, they were shown a second display where the colors could change and had to indicate if the display had changed. Results indicated subjects were more accurate at detecting a change when the items were presented in 3D than 2D. One reason for this improvement could be a reduction in swap errors when making the comparison between the two displays. This could be tested more directly by estimating the proportion of swap errors when items are presented either on a single plane or multiple depth planes. If a reduction in swap errors occurs, this would suggest that depth information is a crucial component in solving multiple forms of visual correspondence.

## Supporting information

S1 Text Detailed model description. (PDF)
