# Supplementary material for: Effect of depth information on multiple-object tracking in three dimensions: A probabilistic perspective
Source: PLoS Comput Biol. 2017 Jul 20;13(7):e1005554. doi: 10.1371/journal.pcbi.1005554 (PMC5519009; doi:10.1371/journal.pcbi.1005554)
Supplement: S1 Text — (PDF) [file pcbi.1005554.s001.pdf]

Effect of depth information on multiple-object tracking in three dimensions: a probabilistic perspective

J. R. H. Cooke, A. C. ter Horst, R. J. van Beers, W. P. Medendorp

Supporting Text S1 - Detailed model description

Contents

1 Detailed model description **1**

1.1 Kalman filter for a single object 1

1.2 Uncertainty in data assignments 3

1.3 Approximate tracking method 4

1.4 Probing the model 5

1 Detailed model description

In this section we derive the tracking algorithm used in the paper by extending the work of Vul et al [1]. This includes the Kalman filter equations for our stimuli and inferring the data assignment vector.

1.1 Kalman filter for a single object

We start by stating the dynamics of our objects in 1D.

$$p_t = p_{t-1} + v_t, \tag{S1}$$

$$v_t = \lambda v_{t-1} - k x_{t-1} + w_t, \tag{S2}$$

$$w_t \sim \mathcal{N}(0, \sigma_w^2) \tag{S3}$$

p_t, v_t, and w_t are the position, velocity, and acceleration at time step t. λ is the damping constant and k is a spring constant. We can express this system using matrices.

$$x_t = A x_{t-1} + w_t, \qquad w_t = \mathcal{N}(0, Q) \tag{S4}$$

$$y_t = x_t + m_t, \qquad m_t = \mathcal{N}(0, R_t) \tag{S5}$$

In which A is the state transition matrix controlling the evolution of the states, x_t is current state at time t consisting of the current position p_t and velocity v_t, w_t is a vector of additive Gaussian noise with covariance Q which alters the states (process noise), y_t is the measurement of the state, which is corrupted by additive noise (measurement noise) with covariance R_t. We extend the 1D dynamics to the

2D case as follows :

$$\mathbf{A} = \begin{bmatrix} 1-k & 0 & \lambda & 0 \\ 0 & 1-k & 0 & \lambda \\ -k & 0 & \lambda & 0 \\ 0 & -k & 0 & \lambda \end{bmatrix},$$

$$\mathbf{x_t} = \begin{bmatrix} p_x \\ p_y \\ v_x \\ v_y \end{bmatrix}, \mathbf{w_t} = \begin{bmatrix} w_{px} \\ w_{py} \\ w_{vx} \\ w_{vy} \end{bmatrix},$$

$$\mathbf{Q} = \begin{bmatrix} \sigma_w^2 & 0 & \sigma_w^2 & 0 \\ 0 & \sigma_w^2 & 0 & \sigma_w^2 \\ \sigma_w^2 & 0 & \sigma_w^2 & 0 \\ 0 & \sigma_w^2 & 0 & \sigma_w^2 \end{bmatrix}, \mathbf{R_t} = \begin{bmatrix} \sigma_{p_x}^2 & 0 & 0 & 0 \\ 0 & \sigma_{p_y}^2 & 0 & 0 \\ 0 & 0 & \sigma_{v_x}^2 & 0 \\ 0 & 0 & 0 & \sigma_{v_y}^2 \end{bmatrix}$$

These were further extended in the 3D case by adding two additional dimensions, corresponding to position and velocity in depth.

We estimated the state of an object with a Kalman filter. To update the state estimate of an object, we first calculate the predicted state $\hat{x}_{t|t-1}$ and covariance of this prediction $G_{t|t-1}$.

$$\hat{x}_{t|t-1} = A\hat{x}_{t-1} \tag{S6}$$

$$G_{t|t-1} = AG_{t-1}A^T + Q \tag{S7}$$

We can then combine this prediction with the observed measurement to estimate the underlying state and compute the covariance of this estimate, the weighting of which is the Kalman gain K_t,

$$\hat{x}_t = \hat{x}_{t|t-1} + K_t(y_t - \hat{x}_{t|t-1}) \tag{S8}$$

$$K_t = G_{t|t-1}(G_{t|t-1} + R_t)^{-1} \tag{S9}$$

$$G_t = (I - K_t)G_{t|t-1} \tag{S10}$$

Because R_t is a function of the underlying state, knowing R_t corresponds to also knowing the underlying state. This is unrealistic in most situations and instead we assumed observers use the

measurement instead of the true state. As such y_t was generated using the true measurement covariance (using x_t) but during tracking R_t was generated using the measurement (using y_t instead). Solving these equations recursively over the entire trial provides the state estimate over the course of a trial.

1.2 Uncertainty in data assignments

As we can see from Eq. S8, updating the state estimate requires computing the error between our measurement and prediction. For a single object this is straightforward as only one measurement exists. For multiple objects this adds the complication of choosing which measurement to assign to which object. One approach is to base our assignment on the likelihood of a measurement arising from an object. Because our model uses linear Gaussian dynamics we can calculate in closed form the likelihood of a measurement arising from a particular object based on its predicted state.

$$p(y_t|\hat{x}_{t|t-1}, G_{t|t-1}, R_t) = \mathcal{N}(y_t; \hat{x}_{t|t-1}, G_{t|t-1} + R_t) \tag{S11}$$

In this equation $\mathcal{N}(y_t; \hat{x}_{t|t-1}, G_{t|t-1} + R_t)$ stands for a Gaussian distribution evaluated at y_t with a mean $\hat{x}_{t|t-1}$ and variance $G_{t|t-1} + R_t$. Intuitively, as the prediction is a Gaussian distribution centered on where the object should be on the next time step we compare the measurement to this prediction with the additional uncertainty introduced by the measurement added.

In order to model how measurements are assigned to objects we defined an assignment vector γ which maps the received measurements into the objects which generated them. For example, the assignment vector $\gamma = [1, 2, 4, 3, 5, 6]$ indicates that the first measurement belongs to object one, the second to object two but the third to object four, and so on. Given this definition of an assignment vector, Eq. S11, and assuming the measurements are independent we can compute the likelihood of a particular assignment vector as the product of the individual likelihoods for each of the measurements,

$$p(\gamma|y_t, \hat{x}_{t|t-1}, G_{t|t-1}, R_t) = \prod_{i=1}^{o} p(y_t(\gamma(i))|\hat{x}(i)_{t|t-1}, G(i)_{t|t-1}, R_t(\gamma(i))) \tag{S12}$$

in which γ is a particular data assignment vector, i is the index of the object, and o is the number of objects.

1.3 Approximate tracking method

In the ideal case, we would compute the state estimates conditional on each assignment vector and then marginalize over all possible assignment vectors. This would require computing 720 state estimates at each time step, which is a considerable computation. We reduced this computation by approximating the final calculation. As we have a closed form expression for the assignment likelihood we need not use all the possible assignment vectors, we instead used a few likely ones to approximate the ideal solution [1, 2]. Formally, this technique is referred to as Rao-Blackwellized particle filtering [3] and enables approximate inference in mixed linear and non-linear state space models.

We approximate the solution using N particles (We used N = 3 for the results presented in the main paper). For brevity we now denote an individual particle as F and j as the particle index . Each particle was represented by an estimated state $\hat{x}_{j,t}$ and the covariance $G_{j,t}$ of the state estimate for each object, which are conditional on previous data associations used by the particle. At the first time step we initiated N identical particles and updated them using the N most likely assignments vectors. At subsequent time steps we computed the log likelihood of each assignment vector given the different particles and added this to the log likelihood of the assignment from the previous step of the corresponding particle. This prevents selecting assignments from particles which were updated with the less likely assignments.

$$L(\gamma|F_{j,t}) = \ln(p(\gamma|\hat{x}_{j,t|t-1}, G_{j,t|t-1}, R_t) + L(\gamma^*|F_{j,t-1}) \tag{S13}$$

where the $*$ indicates the assignment used to update this particle. This leads to weighting each particle by how likely the previous particular assignment was. This calculation produced $720N$ assignment vectors at each time step. We then selected the N most likely from this set and updated the particles using these assignments.

To estimate the state of an object, we then marginalized over the different particles according to their likelihood. The relative weighting of each of the different particles was calculated according to.

$$W_{j,t} = \frac{e^{L(\gamma^*|F_{j,t})}}{\sum_{j=1}^{N} e^{L(\gamma^*|F_{j,t})}} \tag{S14}$$

Which was rewritten for numerical convenience as,

$$W_{j,t} = \frac{e^{L(\gamma^*|F_{j,t})-L(\gamma^*|F_{1,t})}}{\sum_{j=1}^{N} e^{L(\gamma^*|F_{j,t})-L(\gamma^*|F_{1,t})}} \tag{S15}$$

Based on this, the state estimate and its covariance were computed by,

$$\hat{x}_t = \sum_{j=1}^{N} W_{j,t}\hat{x}_{j,t} \tag{S16}$$

$$G_t = \sum_{j=1}^{N} W_{j,t}G_{j,t} \tag{S17}$$

1.4 Probing the model

The above sections provide a way to estimate the state of an object given the uncertain data associations at each time step. We now need to calculate the probability of our subjects response given the model. Ideally, we would compare the estimate at each time step of our model to that of the subjects [4]. However, it is difficult to obtain this estimate at each time step for multiple targets. As such, we employed a probe paradigm with the subjects and with the model.

In the experiment, subjects indicated if a probed object was a target or a non-target. We performed the same test for the model. This was done by drawing a noisy perceptual measurement y from an object's final position (the objects were stationary in this phase so we only used position):

$$y \sim \mathcal{N}(x(i)_f, R(i)_f) \tag{S18}$$

in which y is a noisy perceptual measurement, $x(i)_f$ is the position state of an object i and $R(i)_f$ is the measurement covariance of the position states both at the final time f.

We subsequently calculated the probability this measurement was from a target or a non-target. In this scenario the optimal solution to maximize the probability of being correct is to compute the ratio of posterior probabilities and report the more likely one. That is, to compute the ratio that the probe is a target compared to it being a non-target given a noisy measurement y and indicate the most likely one with response r,

$$d = \frac{p(T|y)}{p(NT|y)} \tag{S19}$$

$$r = \begin{cases} 1 & \text{if } d \geq 1 \\ 0 & \text{otherwise} \end{cases} \tag{S20}$$

$$p(T|y) = \frac{p(y|T)P(T)}{p(y|T)P(T) + p(y|NT)P(NT)} \tag{S21}$$

in which y is the noisy perceptual measurement, T indicates target and NT indicates non-target. We assume that subjects have equal priors on both possibilities, which simplifies Eq. S19 as follows,

$$d = \frac{p(y|T)}{p(y|NT)} \tag{S22}$$

For simplicity we assume the first three objects were targets and the final three non-targets. Object one was also the probe on each simulation. Assuming the objects are independent then we can calculate the likelihood of the probe coming from a target as,

$$p(y|T) = \sum_{i=1}^{3} \sum_{j=1}^{N} W_{j,f} \mathcal{N}(y; \hat{x}(i)_{j,f}, G(i)_{j,f} + R_y) \tag{S23}$$

$$p(y|NT) = \sum_{i=4}^{6} \sum_{j=1}^{N} W_{j,f} \mathcal{N}(y; \hat{x}(i)_{j,f}, G(i)_{j,f} + R_y) \tag{S24}$$

in which y is the noisy measurement and R_y is the measurement covariance of the noisy measurement. $\hat{x}(i)_{j,f}$ and $G(i)_{j,f}$ is the state estimate and covariance according to filter j for a particular object i at the final timestep f.

To obtain the probability of a subjects' response for each condition we simulated 1000 trials and averaged the r produced by the model. This was then used as the probability for a binomial distribution for fitting (see main text).

References

1. Vul E, Frank MC, Tenenbaum JB, Alvarez GA. Explaining human multiple object tracking as resource-constrained approximate inference in a dynamic probabilistic model. Advances in Neural Information Processing Systems 22. 2009; p. 1955–1963.

2. Särkkä S, Vehtari A, Lampinen J. Rao-Blackwellized particle filter for multiple target tracking. Information Fusion. 2007;8(1):2–15. doi:10.1016/j.inffus.2005.09.009.

3. Doucet A, De Freitas N, Murphy K, Russell S. Rao-Blackwellised particle filtering for dynamic Bayesian networks. In: Proceedings of the Sixteenth conference on Uncertainty in artificial intelligence. Morgan Kaufmann Publishers Inc.; 2000. p. 176–183.

4. Bonnen K, Burge J, Yates J, Pillow J, Cormack LK. Continuous psychophysics: Target-tracking to measure visual sensitivity. Journal of Vision. 2015;15(3):14–14. doi:10.1167/15.3.14.
